# The effect of *Helicobacter pylori* infection and different *H*. *pylori* components on the proliferation and apoptosis of gastric epithelial cells and fibroblasts

**DOI:** 10.1371/journal.pone.0220636

**Published:** 2019-08-07

**Authors:** Weronika Gonciarz, Agnieszka Krupa, Krzysztof Hinc, Michał Obuchowski, Anthony P Moran, Adrian Gajewski, Magdalena Chmiela

**Affiliations:** 1 Department of Immunology and Infectious Biology, Institute of Microbiology, Biotechnology and Immunology, Faculty of Biology and Environmental Protection, University of Lodz, Łodz, Poland; 2 Laboratory of Molecular Bacteriology, Intercollegiate Faculty of Biotechnology UG-MUG, Medical University of Gdańsk, Gdansk, Poland; 3 Department of Microbiology, School of Natural Sciences, National University of Ireland Galway, Galway, Ireland; Wayne State University, UNITED STATES

## Abstract

**Background:**

*Helicobacter pylori* colonizes the human gastric mucosa, causing chronic inflammation, peptic ulcers and gastric cancer. A cascade of harmful processes results from the interaction of these bacteria with the gastric epithelium.

**Aim:**

To investigate these processes in terms of upregulation of oxidative stress and cell apoptosis and downregulation of the pro-regenerative activity of cells.

**Methods:**

We employed an *in vivo* guinea pig model at 7 or 28 days postinoculation with *H*. *pylori*, corresponding to an acute or chronic stage of infection, respectively, and an *in vitro* model of guinea pig primary gastric epithelial cells and fibroblasts treated with bacterial components: glycine acid extract (GE), urease subunit A (UreA), cytotoxin-associated gene A protein (CagA) and lipopolysaccharide (LPS). Cells were evaluated for metabolic activity (MTT reduction), myeloperoxidase (MPO) and metalloproteinase (MMP-9) secretion, lipid peroxidation (4-hydroxynonenal (4HNE)), migration (wound healing), proliferation (Ki-67 antigen) and cell apoptosis (TUNEL assay; Bcl-xL, Bax, Bcl-2 expression; caspase 3 cleavage).

**Results:**

Significant infiltration of the gastric mucosa by inflammatory cells *in vivo* in response to *H*. *pylori* was accompanied by oxidative stress and cell apoptosis, which were more intense 7 than 28 days after inoculation. The increase in cell proliferation was more intense in chronic than acute infection. *H*. *pylori* components GE, CagA, UreA, and LPS upregulated oxidative stress and apoptosis. Only *H*. *pylori* LPS inhibited cell migration and proliferation, which was accompanied by the upregulation of MMP-9.

**Conclusions:**

*H*. *pylori* infection induces cell apoptosis in conjunction with increased oxidative stress. Elevated apoptosis protects against deleterious inflammation and neoplasia; however, it reduces cell integrity. Upregulation of cell migration and proliferation in response to injury in the milieu of GE, CagA or UreA facilitates tissue regeneration but increases the risk of neoplasia. By comparison, downregulation of cell regeneration by *H*. *pylori* LPS may promote chronic inflammation.

## Introduction

Gastric epithelial cells form a tight barrier that protects the stomach from the deleterious effects of microbial pathogens by maintaining polarity, adhesion, proliferation and movement [1]. Tight junctions are often a target for many bacterial pathogens, which can cause leaking of this barrier. Thus, the Gram-negative bacterium *Helicobacter pylori*, which colonizes more than 50% of the human population [2–3], disrupts the gastric barrier by the direct action of soluble bacterial components or by their interaction with epithelial cell receptors followed by stimulation of various endogenous signaling pathways [1]. *H*. *pylori* cytotoxin-associated gene A (CagA) protein, which is translocated to epithelial cells via the type IV secretion system, destabilizes the E-cadherin/β-catenin complex in a way that is independent of phosphorylation [4–5]. This leads to the activation of β-catenin, which induces the transformation of gastric epithelial cells [4]. CagA may also interfere with the polarization of the gastric epithelial cell membrane due to the interaction with the protease-activated receptor (PAR1)/mitogen-activated protein kinase (MAPK) pathway, which maintains cell polarization by phosphorylation of microtubule-associated proteins (MAP) [6–8]. During *H*. *pylori* infection, the transcription factors nuclear factor kappa B (NF-κB) and activator protein 1 (AP-1) are activated in response to gastric mucosa damage, followed by stimulation of immune cells to secrete proinflammatory mediators [9–10]. Various immunocompetent cells infiltrating the gastric mucosa, including neutrophils, monocytes, macrophages, T helper 1 lymphocytes (Th1), natural killer cells (NK) and nonimmune gastric epithelial cells, respond to *H*. *pylori*, and soluble compounds of this bacterium, such as urease subunit B (UreB) or outer membrane protein (Oip)A, by the secretion of cytokines and growth factors. They include interleukin **(**IL)-1, IL-6, IL-8, tumor necrosis factor (TNF)-α, granulocyte-macrophage colony-stimulating factor (GM-CSF), monocyte chemoattractant protein (MCP)-1, macrophage migration inhibitory factor (MIF) and IL-23, which enable the differentiation and maturation of dendritic cells (DC) and diminish antigen presentation [9, 11–18]. *H*. *pylori* has developed several mechanisms to endure in the organism. The ability of *H*. *pylori* to survive within phagocytes can be mediated by the bacterial production of catalase [19–2]. Other *H*. *pylori* virulence factors, such as lipopolysaccharide (LPS) and vacuolating cytotoxin (VacA), encoded outside the CagA pathogenicity island (PAI), can cause apoptosis of phagocytes and downregulation of NK cell cytotoxic activity [20–23]. Furthermore, stimulation of the signal transducer and activator of transcription 3 (STAT 3)-dependent pathway in DC through CagA or interaction of dendritic cell-specific adhesion molecule -3 –grabbing nonintegrin (DC-SIGN) with Lewis (Le) sugar determinants in *H*. *pylori* LPS results in the production of anti-inflammatory IL-10 and transforming growth factor (TGF)-®, thus promoting maturation of regulatory T lymphocytes (Treg) that are responsible for silencing the immune response [24–31].

The pathogenesis of *H*. *pylori* infections is associated first with acute and second with chronic inflammatory responses. The infection may result in gastric/duodenal ulcers or gastric cancer development. Excessive inflammation induced by *H*. *pylori* can impair the gastric epithelial barrier and its protective function [32]. Impairment of this function can facilitate the translocation of *H*. *pylori* virulence factors and inflammatory mediators into the circulation, causing the development of a systemic inflammatory response. The mechanisms involved in *H*. *pylori*–related deleterious effects are not fully understood. Potentially, the interactions of bacteria and their soluble components with the gastric barrier can induce pathological processes. However, it is not clear how various soluble *H*. *pylori* components, whose content can change during infection, affect the homeostasis of the gastric barrier. Moreover, it is interesting whether endogenous host factors such as matrix metalloproteinase (MMP)-9, released by proinflammatory cells and potentially by gastric epithelial cells, can influence this process. This protein stimulates cell apoptosis and is involved in the process of cell proliferation.

In this study, we used an *in vivo* model of experimental *H*. *pylori* infection in guinea pigs (*Caviae porcellus*) to show whether there is a correlation between acute (7 days after inoculation) or chronic infection (28 days after inoculation) and inflammation in the gastric mucosa of infected *vs* uninfected animals. Additionally, we analyzed the possible correlation between determinants of oxidative stress, such as myeloperoxidase (MPO), which is released during *H*. *pylori* infection, or 4-hydroxynonenal (HNE), which is a product of lipid peroxidation, and the induction and progression of cell apoptosis, cell migration, and proliferation, which would reflect a pro-regenerative potential of the cells. In our previous study (unpublished data), we showed a correlation between the increased serum concentration of MMP-9 and *H*. *pylori* infection in adult patients with gastritis or coronary heart disease. MMP-9 is a confirmed pro-apoptotic factor and regulator of the cell cycle; therefore, this metalloproteinase was selected for study. Furthermore, we used *in vitro* cellular models of guinea pig primary gastric epithelial cells and fibroblasts to evaluate whether the *H*. *pylori* soluble compounds glycine acid extract (GE), a complex of cell surface antigens; the UreA subunit of urease; CagA; and LPS modulate cell proliferation and apoptosis. The use of recombinant proteins is an artificial approach, since it is unclear whether the different components tested will have an effect similar to live bacteria. Therefore, the results should be interpreted cautiously. *H*. *pylori* causes an island-like infection, so their components can be released into the gastric environment during bacterial cell lysis and then deposited locally in an increased concentration. Therefore, *in vitro* experiments can help to examine the effect of individual bacterial components.

## Materials and methods

### *H*. *pylori* strains and culture conditions

*Helicobacter pylori* reference strain CCUG 17874 (Culture collection, University of Gothenburg, Gothenburg, Sweden), positive for VacA and CagA, was used in this study. *H*. *pylori* bacteria were stored at -80°C in trypticase soy broth (TSB) containing 10% glycerol. Bacteria were cultured under microaerophilic conditions according to a previously described procedure [33].

### *H*. *pylori* stimulators

Glycine acid extract (GE) was purified from the reference *H*. *pylori* strain CCUG 17874 according to previously described methods [34–36] and contained surface antigens of *H*. *pylori*. The LPS concentration in the GE sample was <0.001 EU/ml, as shown by the chromogenic *Limulus amebocyte* lysate test (Lonza, Braine-Alleud, Belgium). It was used for *in vitro* experiments at a concentration of 10 μg/ml.

Recombinant CagA protein (rCagA) was expressed in *E*. *coli* as a fusion protein as previously described [37–38]. CagA protein was a kind gift from Antonello Covacci, IRIS, Siena, Italy), and it was used for *in vitro* experiments at a concentration of 1 μg/ml.

Due to patent claims, the ureA subunit of urease from *H*. *acinonychis* was isolated from the acidic environment of the cheetah stomach and used as a homolog of *H*. *pylori* UreA protein (97% homology). The urease gene was amplified by polymerase chain reaction (PCR) as previously described [39]. UreA was used for *in vitro* experiments at a concentration of 5 μg/ml.

LPS from the reference *H*. *pylori* CCUG 17874 strain was obtained by hot phenol-water extraction and purified by proteinase K, DNase and RNase treatment as previously described [40–41]. *H*. *pylori* LPS and standard *E*. *coli* LPS (serotype O55:B5, Sigma-Aldrich) were used for *in vitro* experiments at a concentration of 25 ng/ml. The antigen concentrations were adjusted experimentally or adopted from previously performed experiments [20, 42–43].

### *H*. *pylori* infection in guinea pigs

Adult (three-month-old), 400–600 g, germ-free male Himalayan guinea pigs were used in the experiments. The animals had been bred in the Animal House at the Faculty of Biology and Environmental Protection, University of Lodz (Poland), kept in cages with free access to drinking water and fed with standard chow. All animal experiments were carried out according to the EU directive and approved by the Local Ethics Committee (LKE9) for Animal Experiments, Medical University of Lódź, Poland, which was established by the Ministry of Science and Higher Education in Poland (Decision 58/ŁB45/2016). The animals were inoculated *per os* with *H*. *pylori* as previously described [33, 42]. During the course of infection until day 7 or 28 after inoculation, animal health was monitored every day based on the following criteria: body weight, water and food intake, behavioral symptoms, skin and fur condition, and diarrhea. Seven or 28 days after the last *H*. *pylori* inoculation, the animals were euthanized by overdosing sodium barbiturate, which was administered intraperitoneally, and gastric tissue was collected for histopathological analysis. Blood samples were processed to obtain serum and then stored at –80°C. *H*. *pylori* infection was confirmed by histopathological staining to visualize *Helicobacter*-like organisms (HLO) and gastric tissue inflammation as well as by polymerase chain reaction (PCR) to detect *cagA* and *ureC* gene sequences according to previously described procedures [33, 42]. Additionally, anti-*H*. *pylori* IgG antibodies in the serum samples were detected by an enzyme-linked immunosorbent assay (ELISA) developed in the laboratory, as previously described [36]. The level of *H*. *pylori* antigens in the stool samples of infected animals was evaluated by commercial ELISA (Immunodiagnostic AG, Bensheim, Germany). In total, 15 animals were used in the study, 5 of which were always controls (noninoculated animals). The remaining 10 animals were infected with *H*. *pylori*, 5 animals per group (n = 5+5). The animals were examined 7 or 28 days after inoculation.

### Preparation of guinea pig gastric tissue for histological and immunohistochemical examination

Gastric tissue was isolated from noninfected animals and infected animals on the 7^th^ or 28^th^ day of infection and processed for histological and immunohistochemical/fluorescence examination as described in detail previously [33, 44]. For histological examination of the inflammatory response and *H*. *pylori* colonization, gastric tissue sections were stained routinely with hematoxylin and eosin. For immunohistochemical/fluorescence analysis, they were stained with selected primary and secondary antibodies (specified below). Cell nuclei were counterstained with 4′,6-diamidine-2-phenylindole (DAPI) as previously described [45]. Histopathological and immunohistochemical imaging was done under a light microscope (YS100 Nikon, Tokyo, Japan), a fluorescence microscope (Zeiss, Axio Scope, A1, Oberkochen, Germany) or confocal microscope (Leica TCS SP, Germany) at a wavelength for FITC (excitation 495 nm, emission 519 nm) or DAPI (358 nm excitation, 461 nm emission) at adjusted magnification [46]. The intensity of fluorescence was measured using ImageJ software version 1.48v (National Institutes of Health, United States). The infiltration of the gastric tissue with granulocytes was evaluated by immunohistochemical staining using the primary rabbit anti-mouse 1A8-Ly-6G antibody conjugated to FITC (Thermo Fisher Scientific, Massachusetts, USA), diluted in 1% BSA/PBS 1:50. The infiltration of the gastric tissue with CD4^+^ or CD8^+^ T lymphocytes was evaluated by staining with mouse anti-pig CD4 or CD8 antibodies (Diaclone, Besancon cedex, France) diluted 1:100 in 1% BSA/PBS, followed by incubation with goat anti-mouse antibody (Jackson ImmunoResearch, Cambridge, Great Britain) diluted in 1% BSA/PBS 1:50. Inflammation was categorized as active on the basis of granulocytes or as chronic on the basis of lymphocyte infiltration. It was graded 0 when no or few inflammatory cells were detected; 1 (mild) when up to 40 cells were detected; 2 (moderate) when up to 60 cells were detected, and 3 (strong) when up to 80 cells were detected. The level of gastric tissue cell proliferation was estimated by immunohistochemical staining of Ki-67 cell cycle antigen with monoclonal anti-Ki-67 antibody (MIB-1 clone, diluted 1:150 in 1% BSA/PBS, Dako, Glostrup, Denmark) and is expressed as a proliferation/labeling index (LI%), reflecting the proportion of Ki-67-positive *vs* -negative epithelial cells, as previously described [33]. For histological examination, the same stomach regions were always imaged in the three experimental groups. The number of stained cells was normalized per high-power field.

### Cell cultures for immunohistochemical examination

Guinea pig primary gastric epithelial cells were prepared according to previously described procedures [44, 47–49].

Guinea pig fibroblasts (CRL-1405) were purchased from the American Type Culture Collection (ATCC, Rockville, Manassas, USA) and cultured in complete RPMI-1640 medium supplemented with 10% heat-inactivated fetal calf serum (FCS) plus 1% penicillin/streptomycin at 37°C in a humidified atmosphere containing 5% CO_2_. The cells were passaged every seven days with 0.25% trypsin/0.02% N,N,N′,N′-ethylenediaminetetraacetic acid (EDTA) (Thermo Fisher Scientific, Waltham, MA, USA) and used for experiments at a concentration of 1·10^5^ cells/ml or 1·10^6^ cells/ml. For immunohistochemical and immunofluorescent staining, both types of cells were fixed with 4% formaldehyde and permeabilized with 0.02% Triton-X-100 (Sigma-Aldrich) in phosphate-buffered saline (PBS). Uncovered space on a glass carrier was blocked by 1 h incubation in 5% bovine serum albumin (BSA)/PBS at room temperature. The cells were then incubated overnight at 4°C with selected primary and secondary antibodies (specified above). The cell nuclei were stained with Hoechst dye (AAT Bioques, Sunnyvale, USA) diluted 1:1000 in PBS or with DAPI (Sigma-Aldrich) (2.5 μg/ml) for 15 min at room temperature. The intensity of fluorescence was measured with a fluorescence microscope (Zeiss, Axio Scope, A1) at the appropriate wavelengths for FITC (excitation 495 nm, emission 519 nm) and Hoechst/DAPI (358 nm excitation, 461 nm emission) at 200 magnification [46] or measured using a Victor2 multifunctional reader (Wallak, Oy, Turku, Finland). Data analysis was done using ImageJ software version 1.48v. Four independent experiments were carried out with three replicates of each variant. Cells were imaged under a fluorescence microscope (Zeiss, Axio Scope, A1, Oberkochen, Germany) or a confocal microscope (Leica TCS SP)

### Cell metabolic activity and proliferation

Primary gastric epithelial cells and fibroblasts were placed in 96-well plates at a concentration of 1×10^6^ cells/well in a volume of 100 ∞l/well and then cultured until adherence, followed by 24 h stimulation with *H*. *pylori* components and estimation of metabolic activity and proliferation. The metabolic activity of cells nonstimulated or stimulated with *H*. *pylori* components for 24 h was evaluated with a colorimetric MTT reduction assay based on converting the tetrazolium yellow dye MTT (3-(4,5-dimethylthiazol-2-yl)-2,5-diphenyltetrazolium bromide, 5 mg/ml; Sigma- Aldrich) by living cells to purple formazan crystals, as previously described [45]. Cell proliferation was detected with a radioactive assay based on the incorporation of tritiated thymidine (^3^H) TdR into cell DNA, as previously described [45], and is expressed as a stimulation index (SI), which shows the relative counts per minute (cpm) ratio, calculated by dividing the cpm for the cells cultivated in culture medium with a stimulator by the cpm for the cells cultured in medium alone. An SI value equal to 1.5 (*cutoff*) or higher was considered a positive result in the proliferation assay. The results from four independent experiments performed in triplicate for each variant of stimulation are shown. Cells were imaged under a light microscope (YS100 Nikon, Tokyo, Japan).

### Migration assays

The migration of both primary guinea pig gastric epithelial cells and fibroblasts was evaluated in a “scratch test” based on the ability of cells to move into a space created by an *in vitro* scratch wound, as previously described [45]. The ability of the cells to heal the wound in response to *H*. *pylori* antigens is expressed as a percentage of cells migrating to the wound zone *vs* the migration of untreated cells. Four independent experiments were carried out in triplicate for each variant of stimulation. Cells were imaged under a light microscope (YS100 Nikon, Tokyo, Japan).

### Determinants of oxidative stress

Myeloperoxidase was detected in gastric tissue homogenates. Briefly, 75 ∞l/well of gastric tissue homogenate was mixed on the plate with 150 ∞l/well of 3,3’5,5’tetrametylobenzydyne (TMB) (20 mM TMB/DMSO in 12.5 ml NaH_2_PO_4_ 270 ∞l/well buffer pH 5.4 with 3 ∞l of 30% H_2_O_2_). After 10 min incubation, at room temperature, 50 ∞l of 1 M H_2_SO_4_ was added to stop the color reaction. Absorbance was read using a Victor 2 reader at a wavelength of 450 nm.

The level of 4HNE as a lipid peroxidation product was evaluated both in cell cultures and tissue specimens. First, tissue specimens (after paraffin removal) and cells cultured in a 96-well plate were subjected to antigen unmasking. After blocking unspecific binding with 5% goat serum in Tris-buffered saline (TBS) for 1 h, the cells and tissue specimens were incubated overnight at 4°C with primary rabbit polyclonal anti-4HNE antibody (Bios, Woburn, Massachusetts, USA) diluted 1:100 (tissue staining) or 1:500 in 1% BSA/PBS (cell staining), followed by incubation with FITC-conjugated secondary goat anti-rabbit antibody (Invitrogen Carlsbad, California, USA) diluted in 1% BSA/PBS 1:200 (tissue staining) or 1:2000 (cell staining). The nuclei were stained with DAPI. The intensity of fluorescence of 4HNE in gastric epithelial cells was estimated using a Victor2 reader at the wavelengths for FITC (excitation 495 nm, emission 519 nm), whereas in tissue samples, the intensity of fluorescence was examined using a fluorescence microscope (Zeiss, Axio Scope, A1) or a confocal microscope (Leica TCS SP) at the wavelengths for FITC (excitation 495 nm, emission 519 nm) and for DAPI (358 nm excitation, 461 nm emission), at adjusted magnification (specified in each figure) [46]. Four independent experiments were carried out in triplicate for each sample tested.

### ELISA for MMP-9

The concentration of total MMP-9 in guinea pig serum samples, gastric tissue homogenates and culture media of primary gastric epithelial cells and fibroblasts stimulated with *H*. *pylori* components for 24 h was measured by an ELISA (MyBiosource, San Diego, CA, USA) with a sensitivity of 0.1 ng/ml as instructed by the manufacturer. Absorbance was estimated using a Victor2 reader at a wavelength of 450 nm. Four independent experiments were carried out in triplicate for each stimulation variant.

### Cell apoptosis

The intensity of apoptosis in guinea pig primary gastric epithelial cells and fibroblasts as well as gastric tissue was evaluated using the TUNEL assay (Cell Meter TUNEL Apoptosis Assay Kit, AAT Bioques, Sunnyvale, USA). Cell nuclei were counterstained with Hoechst diluted 1:1000 in PBS for 15 min at room temperature. Cells with apoptotic changes were visualized as red in a fluorescence microscope (Zeiss, Axio Scope, A1) or in a confocal microscope (Leica TCS SP) at 550 nm excitation and 590 nm emission, at magnification adjusted for the cells or for the gastric tissue (specified in each figure). Four independent experiments were carried out in triplicate for each experimental variant.

To detect the involvement of anti- and proapoptotic proteins in the apoptosis process, gastric epithelial cells and fibroblasts, stimulated previously with *H*. *pylori* antigens for 24 h, gastric tissue specimens from control animals or those infected with *H*. *pylori* were incubated with rabbit monoclonal anti-B-cell lymphoma-extra large (Bcl-xL) antibody (Cell Signaling Technology, Danvers, Massachusetts, USA), anti-Bcl-2-like protein 4 (Bax) antibody (Abbkine, Waltham, MA, UAS), anti-B-cell lymphoma 2 (Bcl-2) antibody (Abbkine, Waltham) or anti-caspase 3 (Cell Signaling Technology, USA) antibody diluted 1:100 or 1:500 in 1% BSA/PBS for tissue or cell staining, respectively. The gastric tissue or primary cells were then treated with the appropriate secondary antibody: FITC-conjugated goat anti-rabbit (Invitrogen Carlsbad, California, USA) diluted 1:2000 in 1% BSA/PBS for the primary cell staining and goat anti-rabbit Alexa Fluor 568-IgG secondary antibody (Invitrogen, USA) diluted 1:200 for tissue staining. Cell nuclei were stained with DAPI. The intensity of fluorescence was detected using a fluorescence microscope (Zeiss, Axio Scope, A1) or a confocal microscope (LeicaTCS SP) at the appropriate wavelengths for FITC (excitation 495 nm, emission 519 nm), for Alexa Fluor 568 (excitation 591 nm, emission 608 nm), and for DAPI (358 nm excitation, 461 nm emission), at magnification adjusted for the cells and for the gastric tissue (specified in each figure). The data were analyzed with ImageJ software version 1.48v. Four independent experiments were carried out in triplicate for each experimental variant.

### Statistical analysis

All values are expressed as the median values with a range. The statistical significance in individual experiments was tested using Statistica 12 PL software with the nonparametric Mann-Whitney U test or Kruskal-Wallis test. Results were considered statistically significant when: *P < 0.05, **P < 0.01, ***P < 0.001.

## Results and discussion

During *H*. *pylori* infection in humans, bacteria adhere to gastric epithelial cells, causing gastric epithelial barrier destabilization, which leads to the development of an inflammatory reaction associated with infiltration of immunocompetent cells in response to IL-6, IL-8 and potentially IL-33 [50–51]. The use of an experimental model of *H*. *pylori* infection in guinea pigs allows us to study a typical outcome of *H*. *pylori* infection [52–53], mostly because guinea pigs are the only laboratory animals with a stomach structure resembling the structure of the human organ. Moreover, guinea pigs are prone to developing inflammation in response to the secretion of IL-8, IL-6 and IL-33 by gastric epithelial and immunocompetent cells [42, 53–54]. In our study, we employed an *in vivo* model of guinea pig experimental *H*. *pylori* infection and *in vitro* cellular models of guinea pig primary gastric epithelial cells and fibroblasts treated with *H*. *pylori* or its soluble components to evaluate the effect of *H*. *pylori* infection and different *H*. *pylori* components on the proliferation and apoptosis of gastric epithelial cells and fibroblasts, as well as the mobility and pro-regenerative potential of these cells.

### Animal study–response to experimental *H*. *pylori* infection

**Fig 1:**
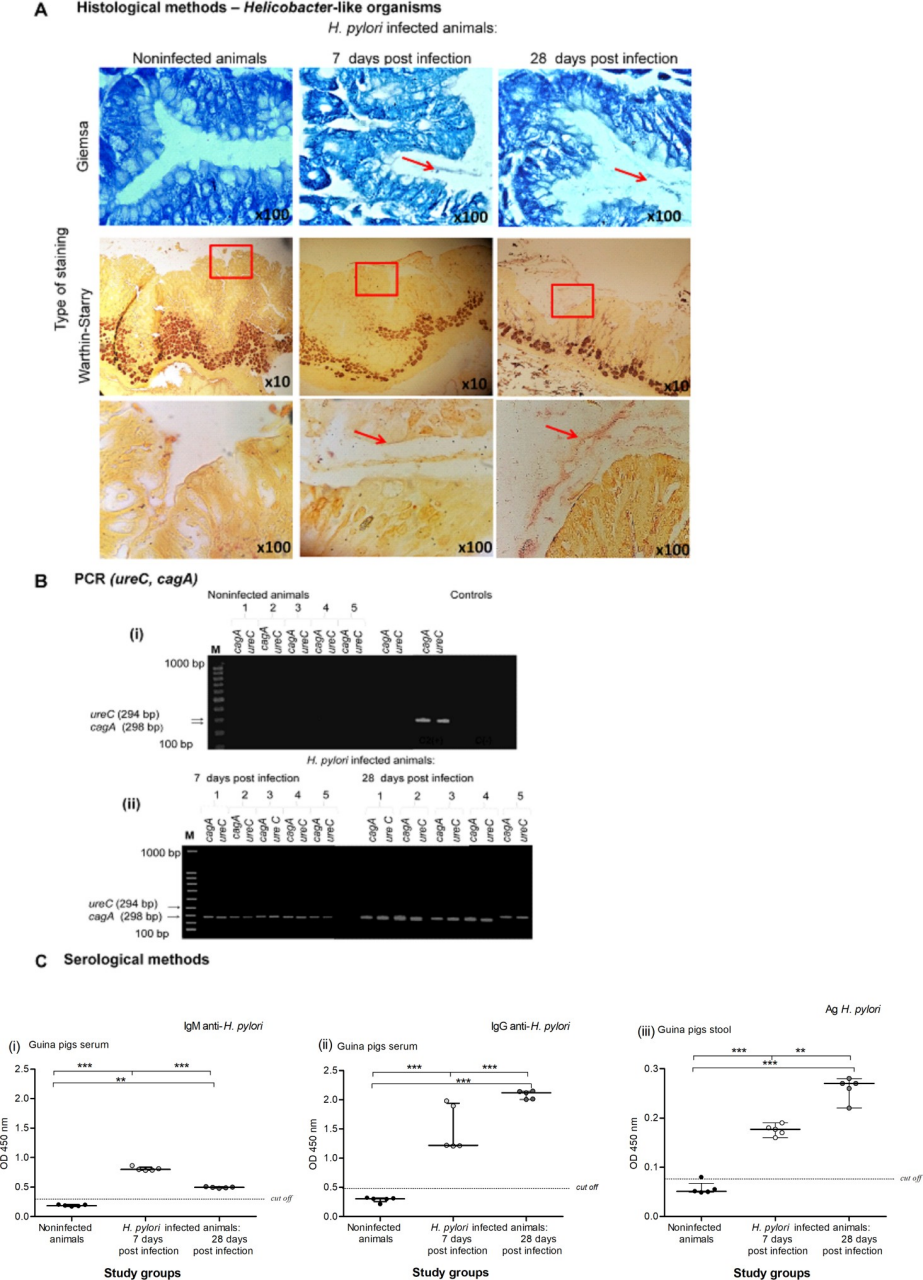
Histological, serological and molecular assessment of *H*. *pylori* infection in an experimental guinea pig model. **(A)** Representative images of gastric tissue sections collected from guinea pigs noninfected or infected with *H*. *pylori* after 7 and 28 days from inoculation, stained with Giemsa and Warthin-Starry method and visualized in a light microscope (YS100, Nikon, Japan). Red arrows and squares indicate the location of *Helicobacter*-like organisms (HLO). **(B)** The electrophoretic separation of the *ureC* and *cagA H*. *pylori* gene amplification products by polymerase chain reaction (PCR) of gastric tissue samples isolated from noninfected or *H*. *pylori* infected guinea pigs after 7 days from inoculation. Samples were run in 1.4% agarose gel in the presence of marker size (M) 100bp and controls: (C [+]—positive control—*H*. *pylori* DNA isolated from *H*. *pylori* suspension at the density 1x10^8^ CFU/ml, C [–]—negative control, reaction mixture lacking DNA). (**i**) Lack of amplification products for *cagA* and *ureC* genes in the gastric tissue samples isolated from control animals. (**ii**) Presence of DNA (amplification products) for CagA (the *cagA* gene—298 bp) and UreC (the *ureC* gene amplification—294 bp) in the gastric tissue samples isolated from animals infected with *H*. *pylori*. **(C)** Serological methods for detection of the serum level of anti-*H*. *pylori* IgM (**i**), anti-*H*. *pylori* IgG (**ii**) and *H*. *pylori* antigens (**iii**) in stool samples collected from guinea pigs noninfected or infected with *H*. *pylori* after 7 and 28 days from inoculation. Five animals per group for control and, 5 per group for infected animals (7 and 28 days after inoculation, 5+5) were examined. The results are presented as median values of four independent experiments performed in triplicates for each experimental variant. Statistical analysis was performed with the nonparametric Kruskal-Wallis test. Statistical significance *P < 0.05, **P < 0.01, ***P < 0.001 was obtained for *H*. *pylori* infected animals (7 and 28 days after inoculation) *vs* control animals; *H*. *pylori* infected animals 7 days *vs* animals 28 days from the last inoculation.

**Fig 2:**
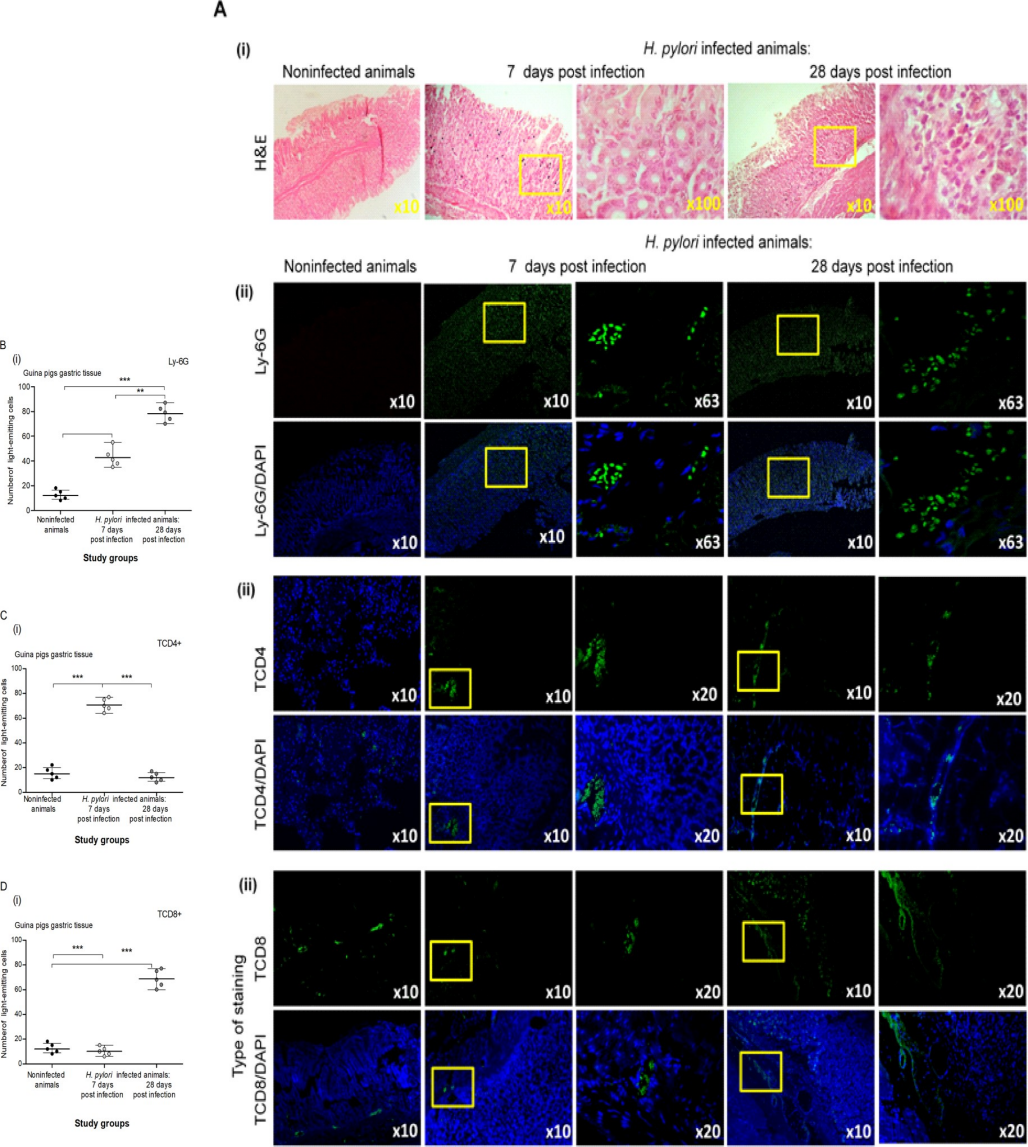
Infiltration of guinea pig gastric mucosa with inflammatory cells in response to *H*. *pylori* infection. **(A)** Representative images of haematoxilin and eosin (H&E) staining of the gastric tissue of guinea pigs noninfected (n = 5) or infected experimentally with *H*. *pylori* after 7 days (n = 5) and 28 days from inoculation (n = 5) photographed in light microscope (YS100 Nikon, Japan). Yellow squares show inflammatory cells infiltration. **(B)** Representative images of neutrophils (Ly-6G marker, FITC—green) in the gastric tissue of noninfected (n = 5) and *H*. *pylori* infected animals (7 and 28 days after inoculation, n = 5+5). The cell nuclei were counterstained with DAPI (blue). Tissue sections were photographed in a confocal microscope (LeicaTCS SP) at wavelengths: FITC 495 nm excitation and 519 nm emission; DAPI 345 nm excitation and 455 nm emission. **(C)** Representative images of CD4+ lymphocytes (FITC—green) in the gastric tissue of noninfected (n = 5) and *H*. *pylori* infected animals (7 and 28 days after inoculation, n = 5+5). The cell nuclei were counterstained with DAPI (blue). Tissue sections were photographed in a confocal microscope (LeicaTCS SP) at wavelengths: FITC 495 nm excitation and 519 nm emission; DAPI 345 nm excitation and 455 nm emission. **(D)** Representative images of CD8+ lymphocytes (FITC—green) in the gastric tissue of noninfected (n = 5) and *H*. *pylori* infected animals (7 and 28 days after inoculation, n = 5+5). The cell nuclei were counterstained with DAPI (blue). Tissue sections were photographed in a confocal microscope (LeicaTCS SP) at wavelengths: FITC 495 nm excitation and 519 nm emission; DAPI 345 nm excitation and 455 nm emission. **(i)** The intensity of fluorescence of Ly6G, CD4+ and CD8+ positive cells in gastric tissue of noninfected (n = 5) and *H*. *pylori* infected animals (7 and 28 days after inoculation, n = 5+5) measured in the Image J software version 1.48v (National Institute of Health, United States). The results are presented as median values of four independent experiments performed in triplicates for each experimental variant. Statistical analysis was performed with the nonparametric Kruskal-Wallis test. Statistical significance *P < 0.05, **P < 0.01, ***P < 0.001 was obtained for *H*. *pylori* infected animals (7 and 28 days after inoculation) *vs* control animals; *H*. *pylori* infected animals 7 days *vs* animals 28 days from the last inoculation.

In our model, the status of *H*. *pylori* infection after 7 and 28 days of inoculation was confirmed by histological, molecular and serological methods, as previously described [33]. For histological analysis, we applied several principles of the Sydney system, a classification of gastritis introduced in 1990 and updated in 1995 [55]. The gastric mucosa of guinea pigs inoculated with *H*. *pylori* was colonized with these bacteria, as shown by Giemsa and Warthin-Starry tissue staining, which were used for visualization of HLO **(Fig 1A).** In the gastric tissue of infected but not control animals, *cagA* and *ureC* sequences were detected by PCR as a 294-base-pair (bp) product and a 298 bp product, respectively **(Fig 1B)**. In a previous study, in the same species, we showed that infection of guinea pigs with *H*. *pylori* was followed by upregulation of mucin 5 (MUC5AC) expression and deposition of Lewis determinants in the gastric mucosa, particularly 7 days after inoculation, which were correlated with enhanced gastric tissue colonization by the bacteria [44]. The infected animals responded to the bacteria by producing anti-*H*. *pylori* IgM and IgG. The level of anti-*H*. *pylori* IgM in serum samples of animals was higher at 7 than at 28 days after inoculation, indicating an acute infection, whereas the animals 28 days after inoculation showed a higher level of anti-*H*. *pylori* IgG than those 7 days after inoculation **(Fig 1C i, ii)**. Similarly, the concentration of *H*. *pylori* antigens in animal stool samples was higher at 28 than at 7 days after inoculation, which confirmed the persistence of infection **(Fig 1C iii).** Histopathological analysis of gastric tissue sections stained with hematoxylin and eosin showed inflammation within the gastric tissue in the *H*. *pylori*-infected animals **(Fig 2A i).** The infection was accompanied by a significant infiltration of granulocytes (Ly-6G positive cells) within 7–28 days after inoculation, which was stronger after 28 days (grade 3) than after 7 days (grade 2) **(Fig 2B i, ii).** The inflammatory response became chronic 28 days after inoculation, as evidenced by the infiltration of lymphocytes, which were located mainly in the submucosa (grade 3) **(Fig 2A i).** However, 7 days after inoculation, a higher number of CD4+ T cells than CD8+ T lymphocytes was detected, whereas 28 days after inoculation, the number of CD8+ T lymphocytes was higher than that of CD4+ T cells **(Fig 2C i, ii and Fig 2D i, ii, respectively)**. These results indicate the engagement of various T lymphocyte subpopulations during the course of infection. Previously, in *vitro* cell cultures carried out with lymphocytes from mesenteric lymph nodes of *H*. *pylori*-infected guinea pigs in the presence of a soluble *H*. *pylori* antigenic complex, the lymphocytes, particularly CD4-positive T cells, proliferated intensively, indicating the domination of this lymphocyte population in the infected guinea pigs four weeks postinfection [42]. In the same study, we analyzed mRNA levels for monocyte-derived cytokines, such as interleukin (IL)-8, IL-1α and interferon gamma (IFN-γ), and for lymphocyte-derived cytokines, including transforming growth factor beta (TGF-β), in leukocyte cell cultures derived from mesenteric lymph nodes of control and *H*. *pylori*-infected guinea pigs [42]. In the monocytes of both uninfected and infected animals, IL-8 mRNA was detected, whereas mRNA for IL-1β was shown only in the monocytes of uninfected animals. TGF-β mRNA was detected in the lymphocytes of control and *H*. *pylori*-infected guinea pigs. Interestingly, a significantly increased level of mRNA for IFN-γ was detected in the lymphocytes from *H*. *pylori* infected compared to noninfected animals and was propagated in the cell cultures only in response to *H*. *pylori* antigens but not in the cell culture medium alone [42]. These results were compatible with the study of Kronsteiner et al. [56], which revealed that *H*. *pylori* infection in guinea pigs induced a predominant systemic response characterized by an increased number of T CD4-positive lymphocytes. Our preliminary results in guinea pigs 60 days after inoculation with *H*. *pylori* confirmed the maintenance of the chronic inflammatory response (unpublished data). The gastric tissue of uninfected animals showed no evidence of increased inflammation **(Fig 2).** Our observations from the present study on the development of an inflammatory process in response to *H*. *pylori* infection are in line with the results obtained by other groups who have studied the development of an inflammatory response in the gastric mucosa of *H*. *pylori*-infected guinea pigs [33, 57–58]. Sjoredn et al. [59] revealed a massive infiltration of mononuclear cells and eosinophils in the gastric mucosa of guinea pigs 3 weeks after inoculation with *H*. *pylori*. Sun et al. [60] showed *H*. *pylori*–induced gastritis in Mongolian gerbils, which was associated with gastric barrier defects extending from the antrum into the corpus over time.

**Fig 3:**
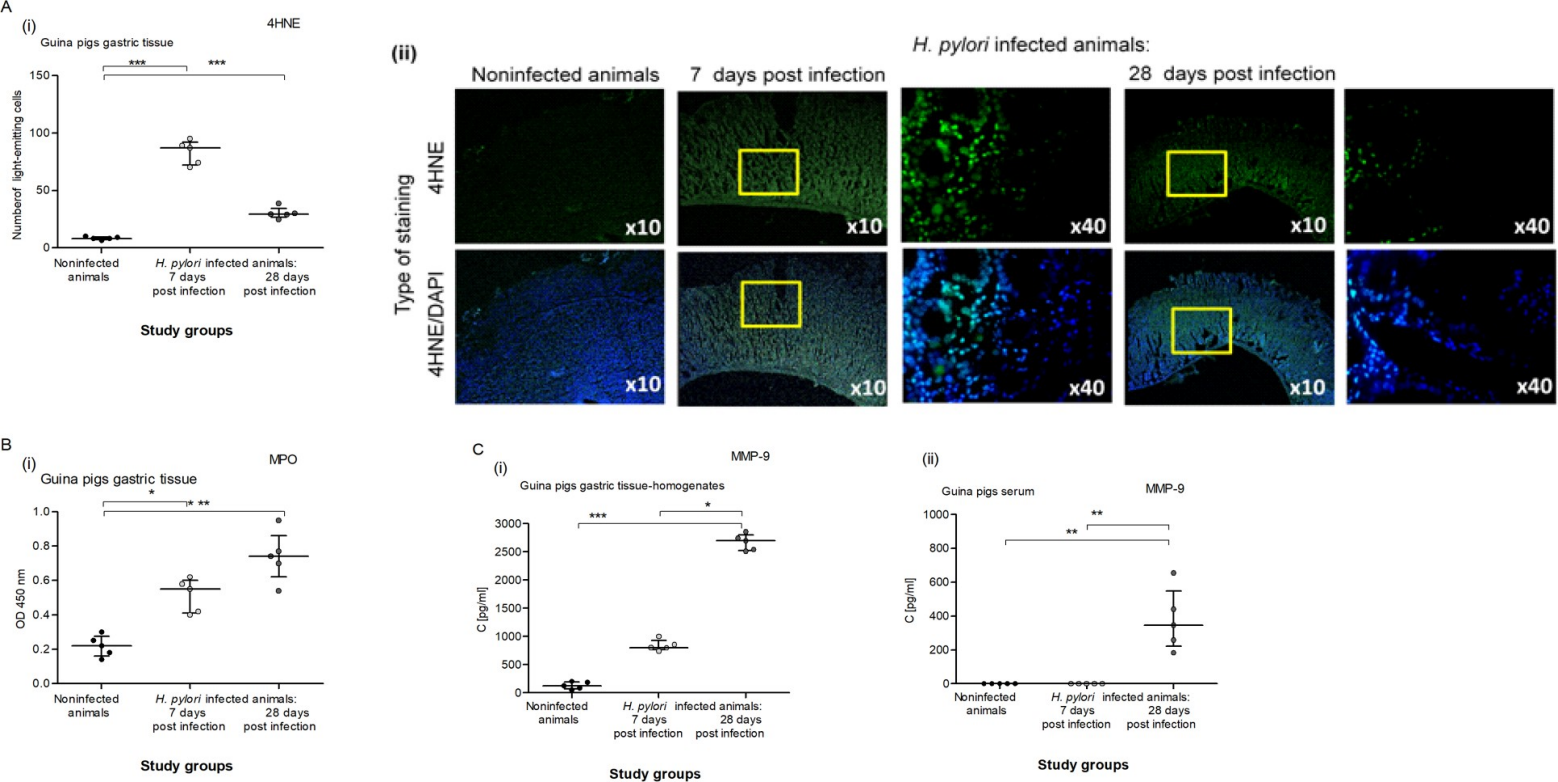
Detection of 4HNE, MPO and MMP-9 in the gastric tissue of *H*. *pylori* infected guinea pigs. **(A)** Oxidative stress assessment in the gastric tissue of guinea pigs noninfected or infected with *H*. *pylori* after 7 and 28 days from inoculation based on the presence of 4-hydroxynonenal (4HNE). (**i**) Fluorescence intensity of 4HNE in the gastric tissue measured using the Image J software version 1.48v (National Institute of Health, United States). **(ii)** Representative images of 4HNE in the gastric tissue visualized in a confocal microscope (LeicaTCS SP) at wavelengths: FITC 495 nm excitation and 519 nm emission; DAPI 345 nm excitation and 455 nm emission. **(B)** Level of myeloperoxidase (MPO) in the gastric tissue homogenates of guinea pigs noninfected or infected with *H*. *pylori* after 7 and 28 days from inoculation. **(C)** Level of metalloproteinase (MMP-9) in gastric tissue homogenates **(i)** or serum **(ii)** of guinea pigs noninfected or infected with *H*. *pylori* after 7 and 28 days from inoculation. Five animals per group for control and, 5 animals per group for infected individuals (7 and 28 days after inoculation, n = 5+5) were examined. The results are presented as median values with a range of four independent experiments performed in triplicates for each experimental variant. Statistical analysis was performed with the nonparametric Kruskal-Wallis test. Statistical significance *P < 0.05, **P < 0.01, ***P < 0.001 was obtained for *H*. *pylori* infected animals (7 and 28 days after inoculation) *vs* control animals; *H*. *pylori* infected animals 7 days *vs* animals 28 days from the last inoculation.

Importantly, oxidative stress induced by *H*. *pylori* components or endogenous factors may contribute to pathophysiologic and histopathological alterations, including autophagy and apoptosis, leading to gastritis and even cancer development [61]. In our study, the *H*. *pylori*-induced inflammatory reaction in the gastric mucosa of infected animals was associated with an increase in oxidative stress, more significantly 7 days than 28 days after inoculation, which we evaluated by monitoring lipid peroxidation in the gastric tissue (4HNE detection) **(Fig 3A)** and MPO concentration **(Fig 3B).** The highest level of 4HNE was observed in animals infected with *H*. *pylori* 7 days after inoculation and remained at an elevated level after 28 days, although it was lower than at 7 days after inoculation. This may suggest an initiation of the antioxidative host response. An increased concentration of MPO was detected in the gastric tissue of infected animals. It was higher at 28 than 7 days after inoculation, which was in line with the increasing number of granulocytes in the gastric epithelium during the course of infection.

Due to the infiltration of the gastric mucosa by neutrophils during *H*. *pylori* infection, we evaluated the local and systemic concentrations of MMP-9 **(Fig 3C).** This matrix metalloproteinase is delivered mainly by activated granulocytes, whose enzymatic activity and secretion of reactive oxygen species intensify oxidative stress in the inflammatory milieu, and by epithelial and endothelial cells in response to various stimuli [62–63]. In our previous study (unpublished data), we showed a correlation between the increased serum concentration of MMP-9 and *H*. *pylori* infection in adult patients with gastritis or coronary heart disease. In this study, the concentration of MMP-9 was evaluated in the gastric tissue homogenates or in the serum of *H*. *pylori*-infected or uninfected guinea pigs **(Fig 3C i, ii).** It was significantly higher in the gastric tissue of infected animals 7 days after inoculation than in uninfected animals and increased further 28 days after inoculation. On the other hand, the level of MMP-9 in the serum of infected animals significantly increased at 28 days, whereas at 7 days, it stayed at the level of control (uninfected animals) **(Fig 3C ii).** Our results suggest that the level of MMP-9 increased first locally and then systemically. MMP-9 catalyzes the normal turnover of extracellular matrix (ECM) macromolecules and can play an important role in repair processes. It is also a confirmed proapoptotic factor and regulator of the cell cycle [64].

**Fig 4:**
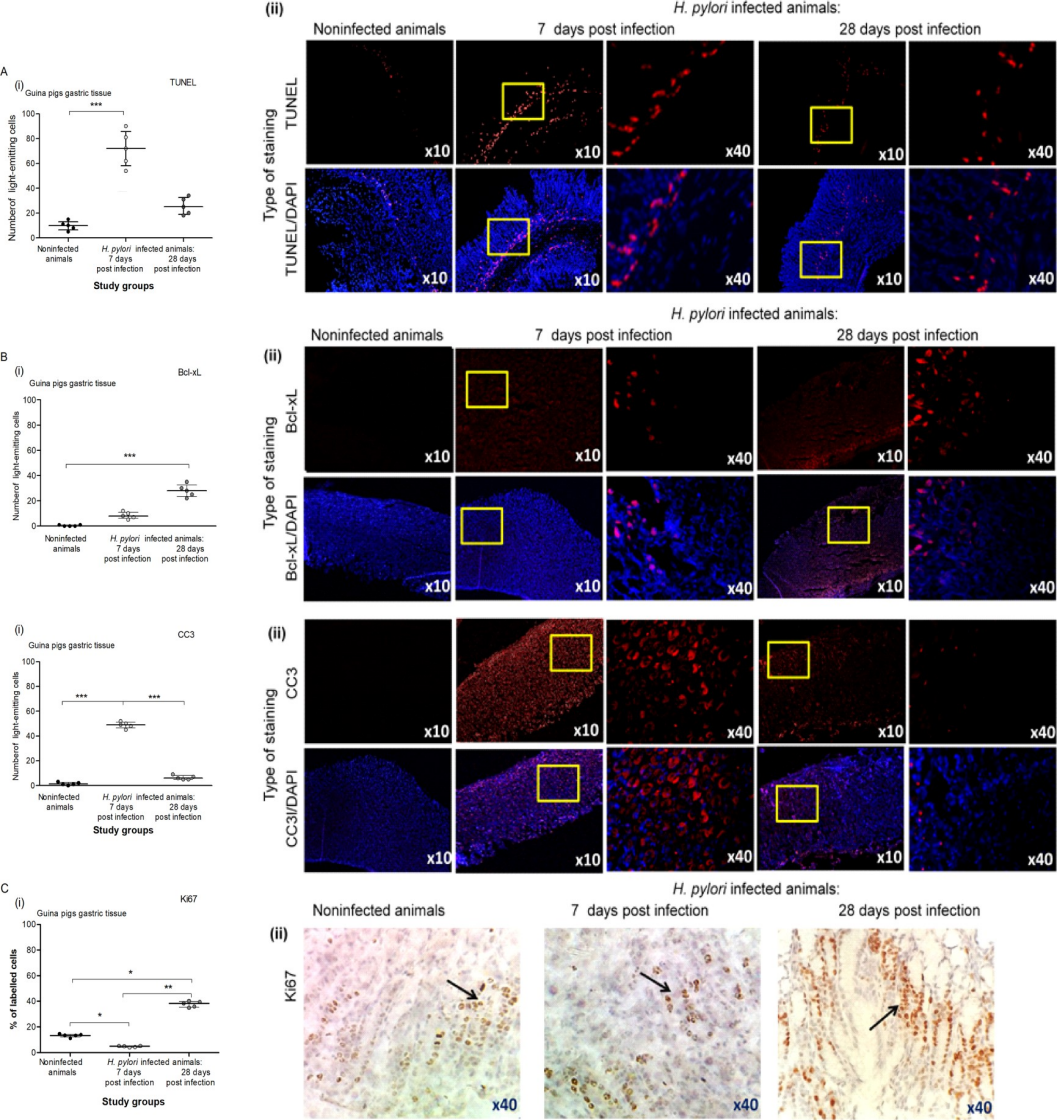
The intensity of apoptosis and proliferation in the gastric tissue of guinea pigs infected with *H*. *pylori*. **(A)** Level of apoptosis was evaluated in the gastric tissue of guinea pigs noninfected or infected with *H*. *pylori* after 7 and 28 days from inoculation. **(i)** Fluorescence intensity of apoptotic cells, detected in the gastric tissue using the TUNEL assay and counterstained with DAPI was measured using the Image J software version 1.48v (National Institute of Health, United States). **(ii)** Representative images of gastric tissue sections stained with the TUNEL assay, counterstained with DAPI and photographed in a fluorescence microscope (Axio Scope A1, Zeiss, Germany) at wavelengths: 550 nm (excitation) and 590 nm (emission) for TUNEL and 345 nm (excitation) 455 nm (emission) for DAPI. **(B)** Level of intensity of anti-apoptotic Bcl-xL in the gastric tissue of guinea pigs noninfected or infected with *H*. *pylori* after 7 and 28 days from inoculation. **(i)** Fluorescence intensity of Bcl-xL in the gastric tissue was measured using the Image J software version 1.48v (National Institute of Health, United States). **(ii)** Representative images of Bcl-xL in the gastric tissue photographed in a confocal microscope (LeicaTCS SP). DAPI was used for nuclear staining. **(C)** Level of intensity of cleaved pro-apoptotic caspase 3 (CC3) in the gastric tissue of guinea pigs noninfected or infected with *H*. *pylori* after 7 and 28 days from inoculation. **(i)** The fluorescence intensity of CC3 in the gastric tissue was measured using the Image J software version 1.48v (National Institute of Health, United States). **(ii)** Representative images of CC3 in the gastric tissue photographed in a confocal microscope (LeicaTCS SP). DAPI was used for nuclear staining. **(D)** Level of Ki-67 (proliferation marker) in the gastric tissue of guinea pigs noninfected or infected with *H*. *pylori* after 7 and 28 days from inoculation. **(i)** Cell proliferation expressed as a labelling index (LI %), indicating the proportion of Ki-67 positive cells (brown labelled-cells) to all epithelial cells, counted using the Image J software program. **(ii)** Representative images of gastric tissue stained for the Ki67. Black arrows show Ki-67 positive cells. Five animals per group for control and, 10 animals per group for infected individuals (n = 5+5) were examined. The results are presented as median values of four independent experiments performed in triplicates for each experimental variant. Statistical analysis was performed in the nonparametric U Mann-Whitney and Kruskal-Wallis tests. Statistical significance *P < 0.05, **P < 0.01, ***P < 0.001 was obtained for *H*. *pylori* infected animals (7 and 28 days after inoculation) *vs* control animals; *H*. *pylori* infected animals 7 days *vs* animals 28 days from the last inoculation.

In humans, *H*. *pylori* infection can modify epithelial cell morphology, causing disruption of tight junctions, induction of cytokine production, epithelial cell proliferation, and an increase in epithelial cell death via apoptosis [65–66]. In our study, we employed the TUNEL assay to show the rate of apoptosis within the gastric mucosa of the studied animals. Our results indicated an increased number of cells undergoing apoptosis in the gastric mucosa of guinea pigs infected with *H*. *pylori* in comparison to noninfected animals. More apoptotic cells were found in the gastric tissue of infected animals after 7 days than after 28 days of infection **(Fig 4A i, ii),** which was compatible with an increased expression of anti-apoptotic Bcl-xL protein in the gastric tissue at 28 days **(Fig 4B i, ii)**. Our observation of cell apoptosis in the TUNEL assay was further confirmed by the detection of an elevated level of cleaved pro-apoptotic caspase 3, which was more pronounced after 7 than after 28 days **(Fig 4C i, ii)**. The severity of apoptotic changes in the gastric tissue may be a response to strong inflammation, especially during an acute phase of infection (in our study, 7 days after the last inoculation), to protect the gastric epithelium against adverse consequences of the inflammatory response. Programmed cell death can reduce microbial infection, separate infected cells from uninfected ones, and alert the host immune system about the danger signals and elevation of inflammatory mediators [67]. Apoptotic cells can induce the production of anti-inflammatory mediators such as transforming growth factor (TGF), prostaglandin E2, and platelet activating factor by macrophages [68]. Many factors, including infectious agents and their components as well as signal proteins from several signaling pathways activated during the inflammation process, are involved in the regulation of cell apoptosis. Usually, the clearance of apoptotic cells by macrophages and epithelial cells downregulates the inflammatory response; however, *H*. *pylori* show the ability to diminish the phagocytic activity of neutrophils and macrophages using a variety of strategies [19–20, 69]. This disorder can result in the failed clearance of apoptotic cells and prolongation of the inflammatory response, which was shown for the first time in pulmonary diseases [70]. Bimczok et al. [71] revealed that epithelial cells undergoing apoptosis in response to live *H*. *pylori* were not eliminated by human gastric mononuclear phagocytes, even though they were under the influence of TNF-α, released by macrophages and highly expressed in *H*. *pylori-*infected gastric mucosa in comparison to control cells [71]. In the present study, an intense infiltration of the gastric mucosa in *H*. *pylori*-infected animals by lymphocytes may suggest their role in the apoptosis of gastric epithelial cells, which was also suggested by other authors [72–73].

The loss of epithelial cells within the gastric tissue during *H*. *pylori* infection requires enhancement of the proliferative activity of gastric epithelial cells. In this study, the Ki-67 protein was a marker of cell proliferation in the gastric tissue of control or infected animals. Our results indicated that epithelial cells proliferated more intensively in the chronic phase of infection, suggesting the pro-regenerative potential of these cells **(Fig 4C i, ii)**. However, the elevated proliferation activity of gastric epithelial cells in response to *H*. *pylori* may increase the risk of mutations, which can be relevant for gastric cancer development [74].

### Cell study–response to soluble components of *H*. *pylori*

**Fig 5:**
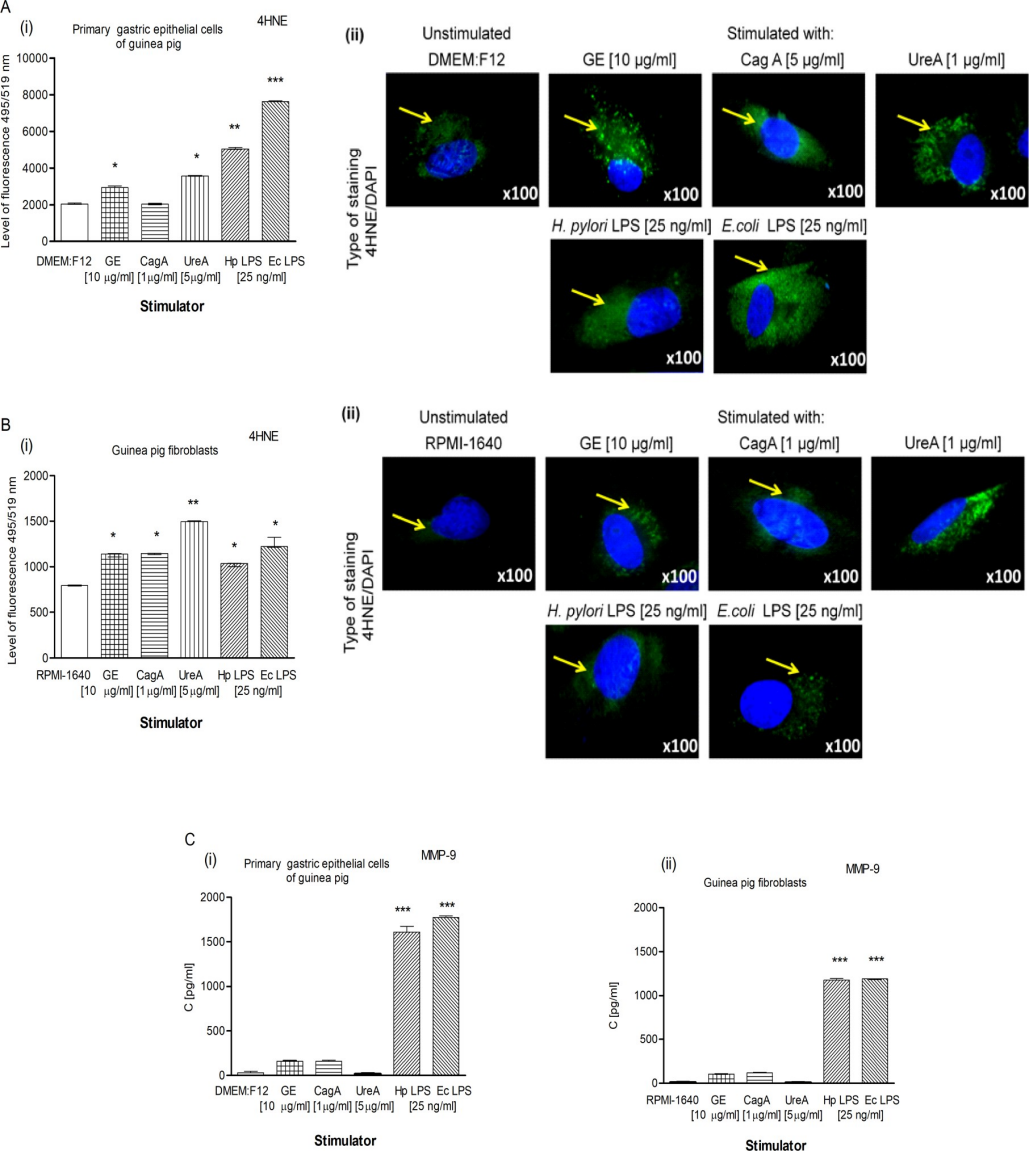
Determinants of oxidative stress in the guinea pig primary gastric epithelial cells and fibroblasts. Oxidative stress assessment based on the production of 4HNE estimated in primary gastric epithelial cells (**A)** and fibroblasts (**B),** nontreated or treated for 24h with *H*. *pylori* antigens: glycine acid extract (GE) (10 μg/ml), cytotoxin associated gene A (CagA) protein (1 μg/ml), urease A subunit (Ure A) (5 μg/ml) and *H*. *pylori* (Hp) or *E*. *coli* (Ec) lipopolysaccharide (LPS) (25 ng/ml). **(i)** Fluorescence intensity of 4HNE in the epithelial cells or fibroblasts measured using the Image J software version 1.48v (National Institute of Health, United States). **(ii)** Representative images of primary gastric epithelial cells and fibroblasts stained for 4HNE and photographed in a fluorescence microscope (Axio Scope A1, Zeiss, Germany). DAPI was used for nuclear staining. The yellow arrows show 4HNE localization in cells. **(C)** Production of MMP-9 by primary gastric epithelial cells **(i)** and fibroblasts **(ii)** in response to *H*. *pylori* compounds detected using the ELISA assay. The results are presented as median values of four independent experiments performed in triplicates for each experimental variant. Statistical analysis was performed in the nonparametric U Mann-Whitney test with significance for *P < 0.05, **P < 0.01, ***P < 0.001 obtained for unstimulated cells *vs* cells treated with *H*. *pylori* antigens.

*H*. *pylori* bacteria are the source of a variety of soluble or cell surface-bound virulent factors, which, by associating with host cells, influence disease development [22,75–76]. We employed primary gastric epithelial cells and fibroblasts to study *in vitro* the effects of well-characterized *H*. *pylori* antigens such as GE, CagA, UreA and LPS on the condition of the epithelial cell barrier. Importantly, both types of cells used in the experiments were developed from a guinea pig, which made the model more relevant to an *in vivo* model of *H*. *pylori* infection used in this study. Fibroblasts, which are present in the subepithelial mucosa, can be targeted by *H*. *pylori* compounds, which leak through epithelial lesions or are transferred there via the epithelium [77]. Furthermore, fibroblasts are involved in the wound healing process and inflammation by responding to pro-inflammatory cytokines [78–79]. Our results showed a significant increase in 4HNE due to lipid peroxidation in primary gastric epithelial cells as well as fibroblasts in response to stimulation with *H*. *pylori* components **(Fig 5A i, ii and Fig 5B i, ii)**. Interestingly, the level of 4HNE in gastric epithelial cells upon stimulation with *H*. *pylori* LPS was almost as intensive as in response to *E*. *coli* endotoxin (positive control). Moreover, the increased amount of 4HNE in gastric epithelial cells and fibroblasts stimulated with *H*. *pylori* LPS was parallel with the MMP-9 release to the culture supernatants **(Fig 5C i, ii),** which may also be also potentially reflected *in vivo*. However, there was no significant difference in the MMP-9 concentration between uninfected cells and the cells treated with GE, CagA or UreA. This may suggest that *in vivo* during *H*. *pylori* infection, MMP-9 can be delivered by the immunocompetent cells infiltrating the gastric mucosa, particularly granulocytes.

**Fig 6:**
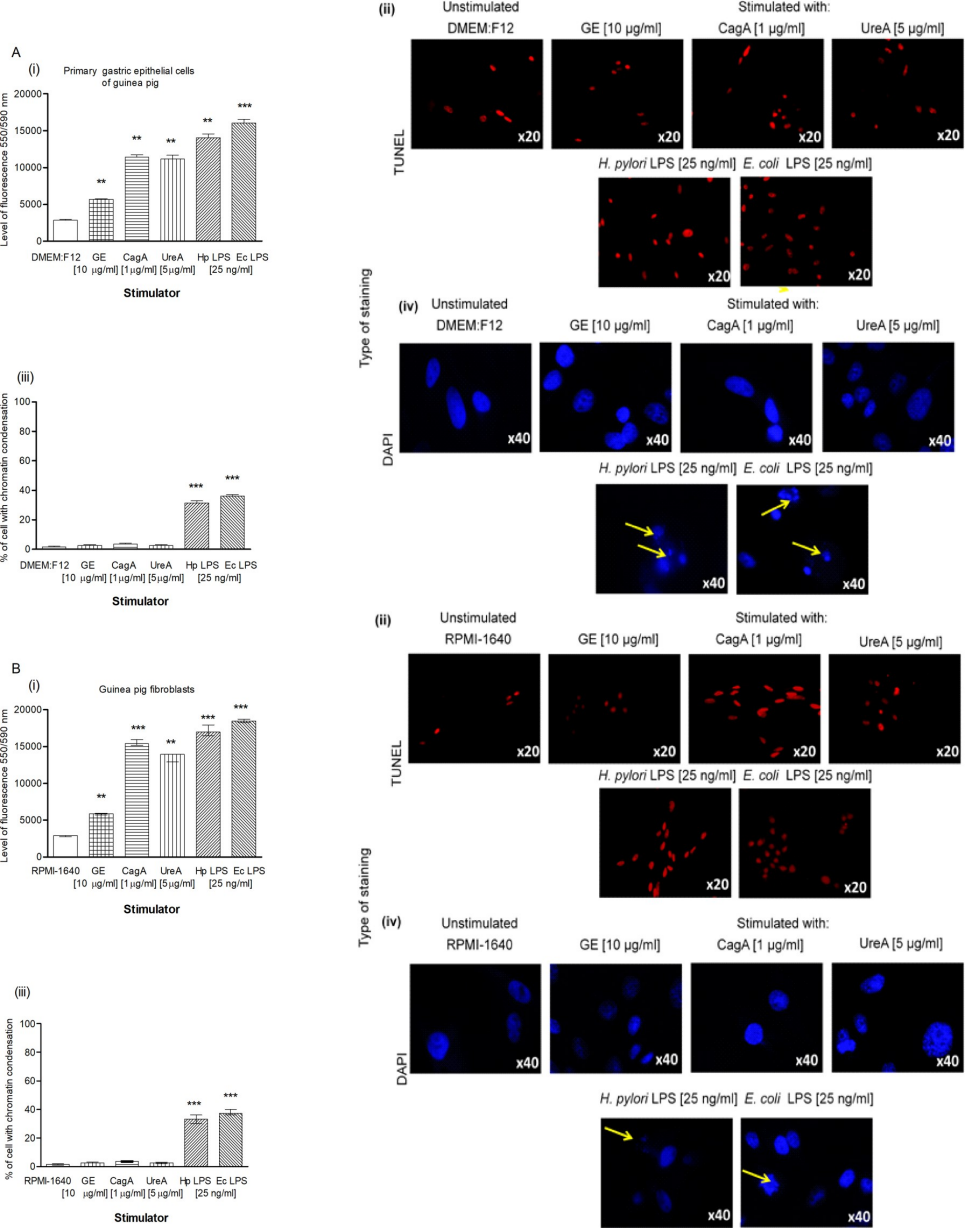
Detection of apoptosis intensity in gastric epithelial cells and fibroblasts treated with *H*. *pylori* components. Cell apoptosis assessed by the TUNEL assay and nuclear staining with DAPI was estimated in cell cultures of primary gastric epithelial cells **(A)** and fibroblasts (**B)**, unstimulated or stimulated for 24h with bacterial antigens: glycine acid extract (GE) (10 μg/ml), cytotoxin associated gene A (CagA) protein (1 μg/ml), urease A subunit (Ure A) (5 μg/ml) and *H*. *pylori* (Hp) or *E*. *coli* (Ec) lipopolysaccharide (LPS) (25 ng/ml). **(i)** Intensity of fluorescence of primary epithelial cells and fibroblasts stained in the TUNEL assay and counted using the Image J software version 1.48v (National Institute of Health, United States). **(ii)** Representative images of primary epithelial cells or fibroblasts stained in the TUNEL assay (red nuclei) photographed in a fluorescence microscope (Axio Scope A1, Zeiss, Germany) at wavelengths: 550 nm (excitation) and 590 nm (emission). **(iii)** The percentage of primary epithelial cells and fibroblasts stained with DAPI and indicated DNA damage. **(iv)** Representative images of primary epithelial cells and fibroblasts stained with DAPI (blue nuclei) and photographed in a fluorescence microscope (Axio Scope A1, Zeiss, Germany) at wavelengths: 345 nm (excitation) 455 nm (emission). Yellow arrows show disintegration of chromatin. The results are presented as median values of four independent experiments performed in triplicates for each experimental variant. Statistical analysis was performed in the nonparametric U Mann-Whitney test with significance for *P < 0.05, **P < 0.01, ***P < 0.001 obtained for unstimulated cells *vs* cells treated with *H*. *pylori* antigens.

**Fig 7:**
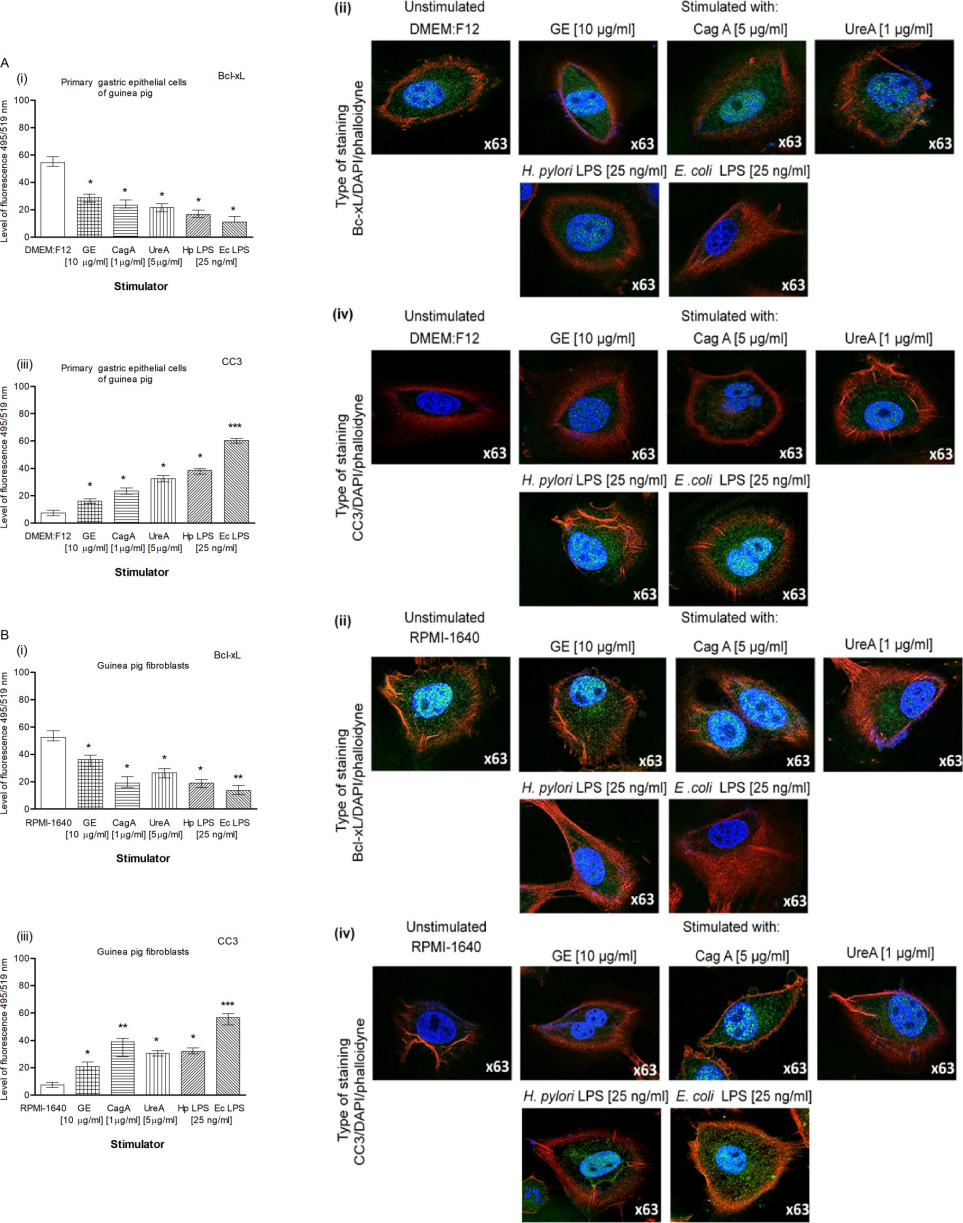
The Bcl-xL and CC3 in gastric epithelial cells and fibroblasts treated with *H*. *pylori* components. Level of anti-apoptotic Bcl-xL and cleaved pro-apoptotic caspase 3 (CC3) in cell cultures of primary gastric epithelial cells **(A)** and fibroblasts **(B)** unstimulated or stimulated for 24h with bacterial antigens: glycine acidextract (GE) (10 μg/ml), cytotoxin associated gene A (CagA) protein (1 μg/ml), urease A subunit (Ure A) (5 μg/ml), *H*. *pylori* (Hp) and *E*. *coli* (Ec) lipopolysaccharide (LPS) (25 ng/ml). Level of intensity of Bcl-xL**(i)** and CC3 **(iii)** in primary epithelial cells and fibroblasts was measured using the Image J software version 1.48v (National Institute of Health, United States). Representative images of primary epithelial cells and fibroblasts stained for Bcl-xL **(ii)** and CC3 **(iv)** and photographed in a confocal microscope (LeicaTCS SP). DAPI was used for nuclear staining and phalloidine for cytoskeleton staining. The results are presented as median values of four independent experiments performed in triplicates for each experimental variant. Statistical analysis was performed in nonparametric U Mann-Whitney test with significance for *P < 0.05, **P < 0.01, ***P < 0.001 obtained for unstimulated cells *vs* cells treated with *H*. *pylori* antigens.

**Fig 8:**
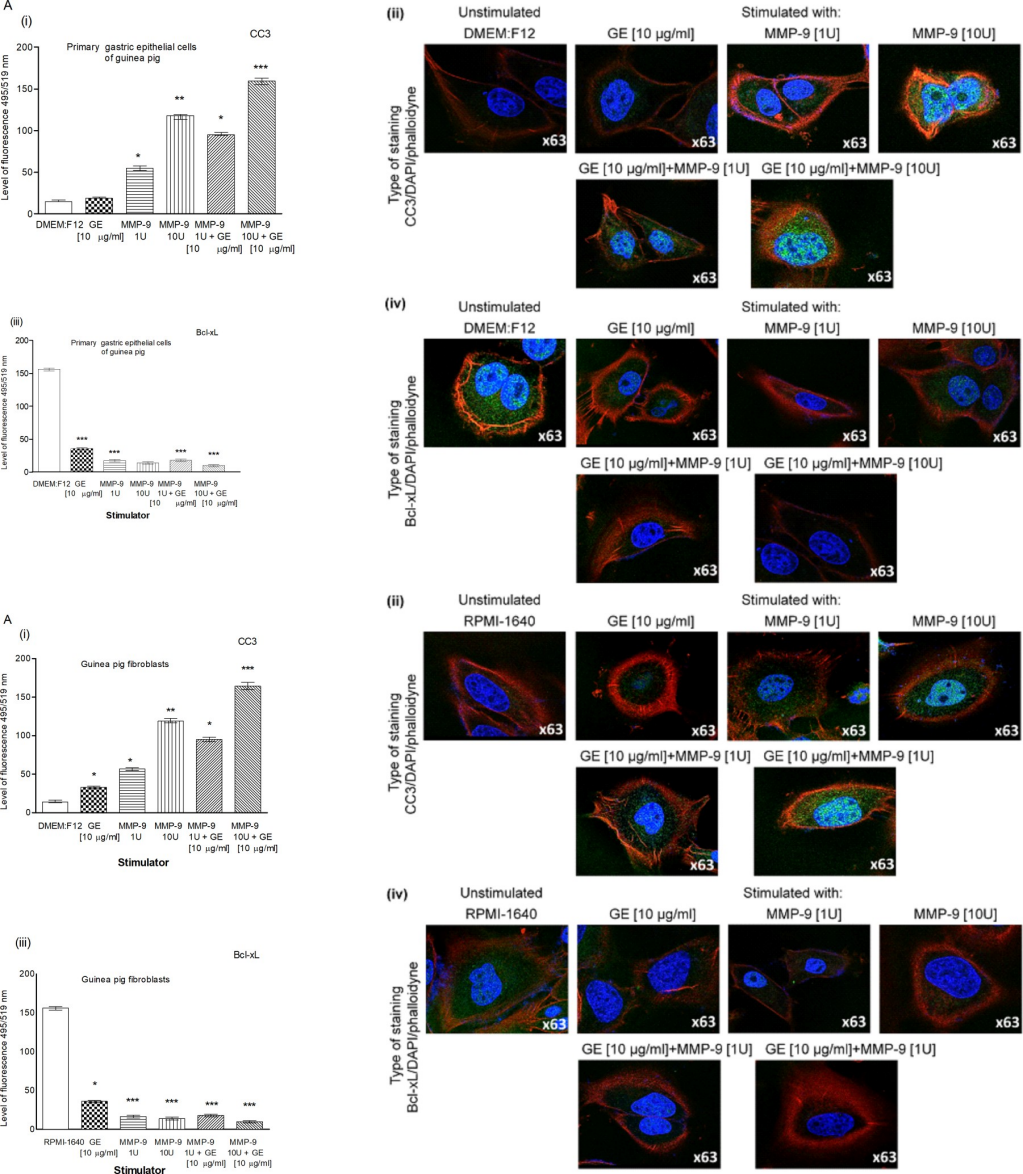
The Bcl-xL and CC3 in gastric cells and exposed to *H*. *pylori* components and/or metalloproteinase (MMP)-9. Level of anti-apoptotic Bcl-xL and cleaved pro-apoptotic caspase 3 (CC3) in cell cultures of primary gastric epithelial cells **(A)** and fibroblasts **(B)** unstimulated or stimulated for 24h with: glycine acid extract (GE) (10 μg/ml), MMP-9 (1 U– 7.5 ng/ml), MMP-9 (10 U– 75 ng/ml), MMP-9 (1 U– 7.5 ng/ml) + GE (10 μg/ml) and MMP-9 (10 U– 75 ng/ml) + GE (10 μg/ml). Level of intensity of Bcl-xL **(i)** and CC3 **(iii)** in primary epithelial cells and fibroblastsmeasured using the Image J software version 1.48v (National Institute of Health, United States). Representative images of primary epithelial cells and fibroblasts stained for Bcl-xL **(ii)** and CC3 **(iv)** and photographed in a confocal microscope (LeicaTCS SP). DAPI was used for nuclear staining and phalloidine for cytoskeleton staining. The results are presented as median values of four independent experiments performed in triplicates for each experimental variant. Statistical analysis was performed in nonparametric U Mann-Whitney test with significance for *P < 0.05, **P < 0.01, ***P < 0.001 obtained forunstimulated cells *vs* cells treated with *H*. *pylori* antigens.

**Fig 9:**
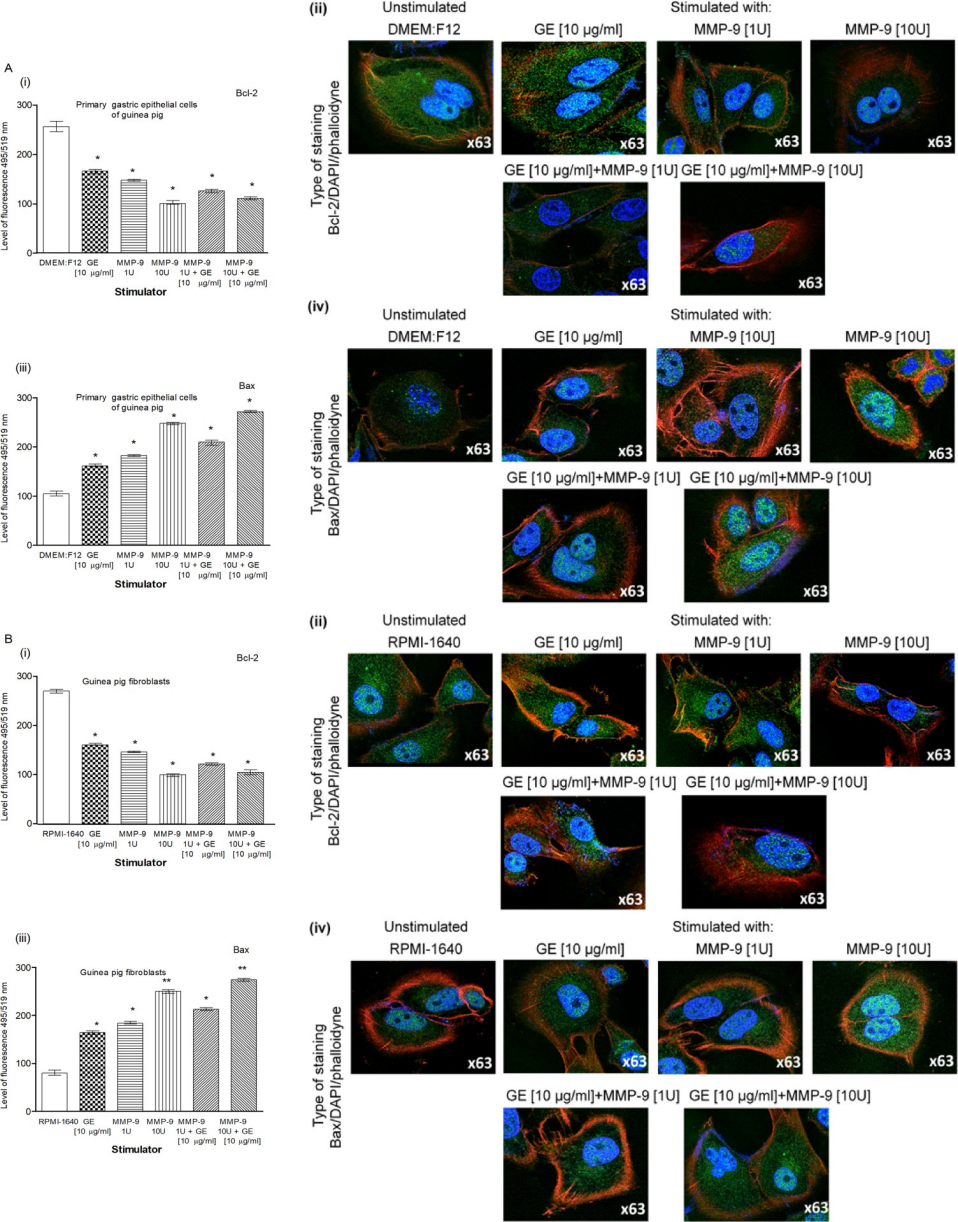
The Bax and Bcl -2 in gastric cells exposed to *H*. *pylori* components and/or metalloproteinase (MMP-9). Level of pro-apoptotic Bax and anti-apoptotic Bcl-2 in cell cultures of primary gastric epithelial cells **(A)** and fibroblasts **(B)** unstimulated or stimulated for 24h with: glicyne acid extract (GE) (10 μg/ml), MMP-9 (1 U– 7.5 ng/ml), MMP-9 (10 U– 75 ng/ml), MMP-9 (1 U– 7.5 ng/ml) + GE (10 μg/ml) and MMP-9 (10 U– 75 ng/ml) + GE (10 μg/ml). Level of intensity of Bax **(i)** and Bcl-2 **(iii)** in primary epithelial cells and fibroblasts measured using the Image J software version 1.48v (National Institute of Health, United States). Representative images of primary epithelial cells and fibroblasts stained for Bax **(ii)** and Bcl-2 **(iv)** and photographed in a confocal microscope (LeicaTCS SP). DAPI was used for nuclear staining and phalloidine for cytoskeleton staining. The results are presented as median values of four independent experiments performed in triplicates for each experimental variant. Statistical analysis was performed in nonparametric U Mann-Whitney test with significance for *P < 0.05, **P < 0.01, ***P < 0.001 obtained for unstimulated cells *vs* cells treated with *H*. *pylori* antigens.

**Fig 10:**
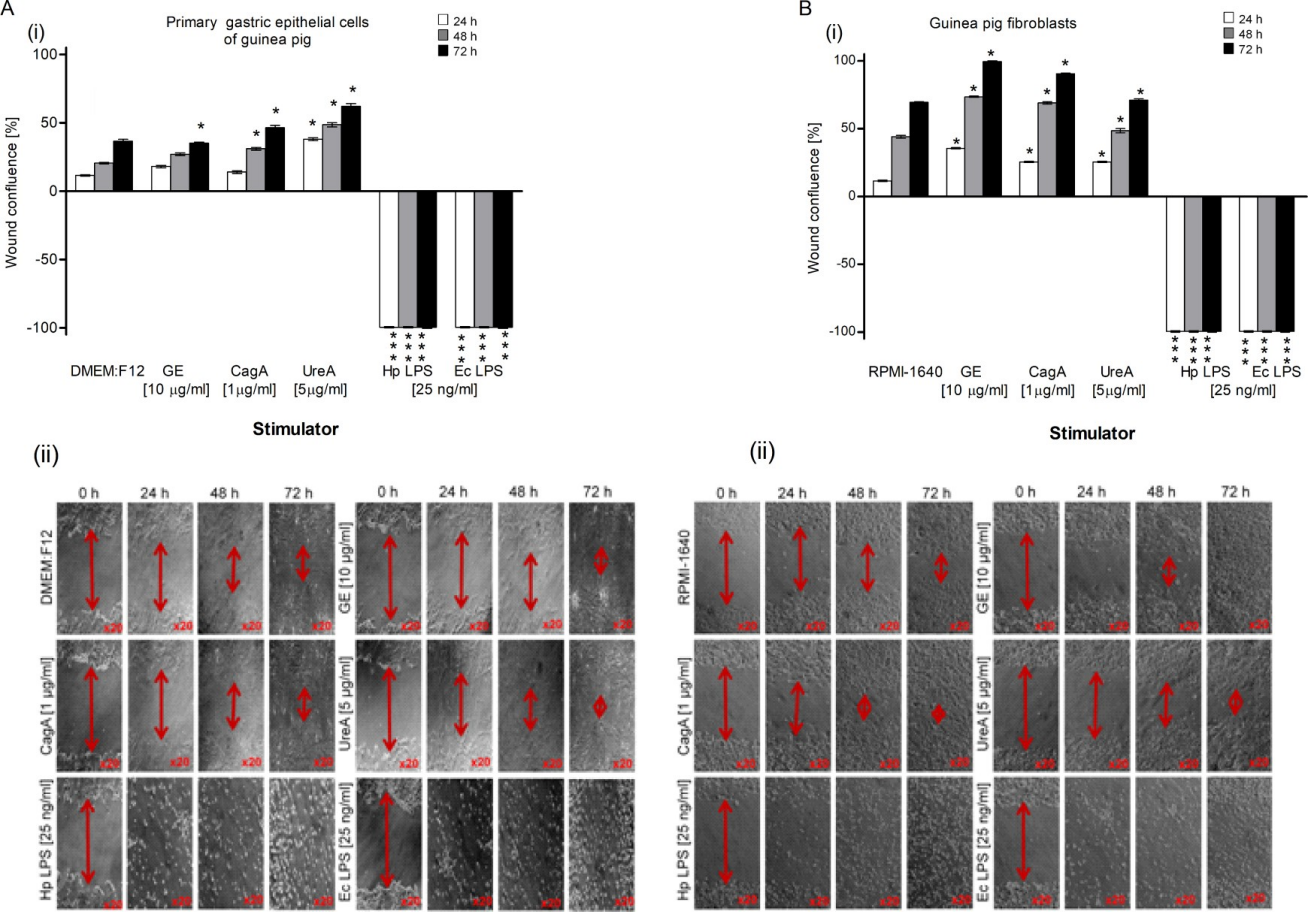
Total cell growth of gastric epithelial cells and fibroblasts exposed to *H*. *pylori* components. Total cell growth of primary gastric epithelial cells (**A)** and fibroblasts (**B)** unstimulated or stimulated with bacterial antigens: glycine acid extract (GE) (10 μg/ml), cytotoxin associated gene A (CagA) protein (1 μg/ml), urease A subunit (Ure A) (5 μg/ml) and *H*. *pylori* (Hp) or *E*. *coli* (Ec) lipopolysaccharide (LPS) (25 ng/ml) detected based on (^3^H)-thymidine incorporation into cellular DNA **(i)** or MTT reduction assay **(ii). (i)** Proliferation of primary gastric epithelial cells or fibroblasts shown as the graph presenting a stimulation index (SI) calculated by dividing the radioactivity counts (cpm/min), for the cell cultures in the presence of a stimulus, by the counts for control cell cultures in culture medium alone. The results are shown as median value with a range. The results obtained for six independent experiments, performed in triplicates for each experimental variant are presented. **(ii)** Reduction of MTT salt by primary epithelial cells and fibroblasts shown as a graph presenting the average percentage of cells (unstimulated and stimulated with *H*. *pylori* antigens) able to reduce MTT. Data represent the average values of four independent experiments performed in triplicates for each experimental variant. Statistical analysis was performed in the nonparametric U Mann-Whitney test with significance for *P < 0.05, **P < 0.01, ***P < 0.001 obtained for unstimulated cells *vs* cells treated with *H*. *pylori* antigens.

**Fig 11:**
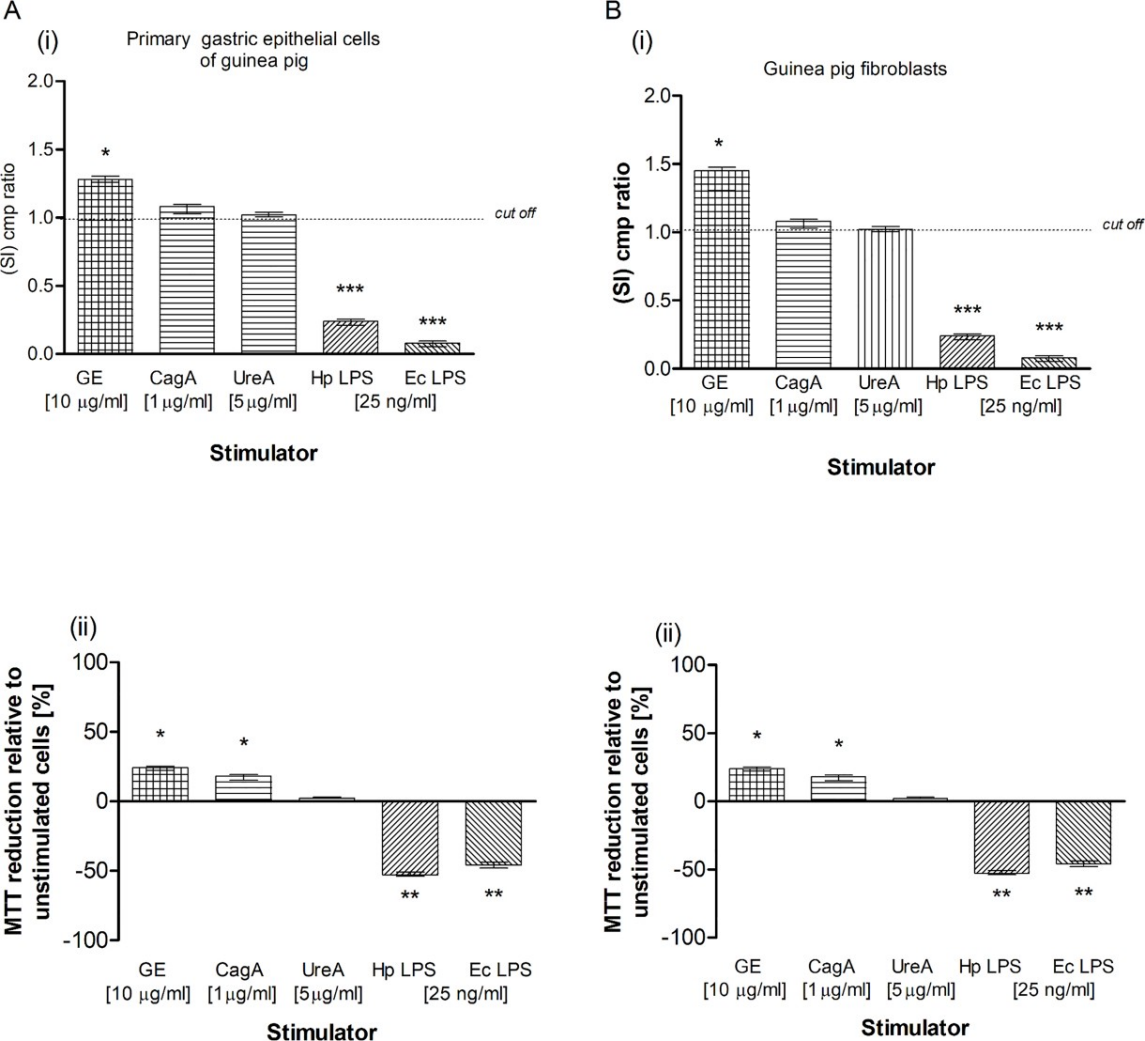
The effectiveness of migration of gastric epithelial cells and fibroblasts exposed to *H*. *pylori* components. The migration of primary gastric epithelial cells **(A)** and fibroblasts **(B)** unstimulated or treated with: glycine acid extract (GE) (10 μg/ml), cytotoxin associated gene A (CagA) protein (1 μg/ml), urease A subunit (Ure A) (5 μg/ml), *H*. *pylori* (Hp) or *E*. *coli* (Ec) lipopolysaccharide (LPS) (25 ng/ml) and detected in a scratch assay. **(i)** The migration of primary epithelial cells and fibroblasts shown as a graph presenting wound confluence [%] (average wound size against time). The results are presented as median values of four independent experiments performed in triplicates for each experimental variant. Statistical analysis was performed in the nonparametric U Mann-Whitney test with significance for *P < 0.05, **P < 0.01, ***P < 0.001 obtained for unstimulated cells *vs* cells treated with *H*. *pylori* antigens (according to the used stimulators). **(ii)** Phase-contrast microscopy images of a scratch assay. Pictures were taken at the indicated time points and the extent of wound closure for each treatment variant is shown (red arrows). Representative images of each time point for both types of cells are shown.

Our results obtained by the TUNEL assay showed an increased number of primary gastric epithelial cells **(Fig 6A, i, ii)** and fibroblasts **(Fig 6B i, ii)** undergoing apoptosis in response to the treatment with GE, CagA, UreA or LPS of *H*. *pylori*, and the LPS showed the strongest pro-apoptotic activity, similar to *E*. *coli* LPS. In cultures treated with *H*. *pylori* LPS or standard *E*. *coli* LPS, apoptosis was irreversible, as confirmed by the chromatin condensation and disintegration of cell nuclei in DAPI staining **(Fig 6A iii, iv and Fig 6B, iii, iv).** To confirm our observation that *H*. *pylori* components upregulated apoptosis of both types of cells, as detected by the TUNEL stain, we analyzed cleaved pro-apoptotic caspase 3 (CC3) and Bax as well as anti-apoptotic Bcl-xL and Bcl-2 expression in cells. As shown in **Fig 7A i, ii** and **Fig 7B i, ii**, the expression of anti-apoptotic Bcl-xL was downregulated, whereas the expression of pro-apoptotic CC3 increased in gastric epithelial cells and fibroblasts treated with *H*. *pylori* components. Because gastric epithelial cells and fibroblasts stimulated with *H*. *pylori* LPS released a significant amount of MMP-9 into the culture environment and this enzyme is a well-recognized inducer of cell apoptosis [64], we studied its role in *H*. *pylori*-induced cell survival regulation. The addition of exogenous MMP-9 to the cultures of gastric epithelial cells and fibroblasts, which were carried out with *H*. *pylori* components, resulted in a more intense production of CC3, a diminished expression of anti-apoptotic Bcl-xL (**Fig 8A and 8B)** and anti-apoptotic Bcl-2 protein, and an increased expression of pro-apoptotic Bax protein **(Fig 9A and 9B)**. Pierzchalski et al. [80] suggested that only live *H*. *pylori* bacteria were capable of inducing caspase-3-dependent apoptosis in gastric epithelial cells. Our results showed not only irreversible apoptosis of gastric epithelial cells and fibroblasts exposed to *H*. *pylori* LPS but also a reduced proliferative activity of cells measured in the MTT reduction assay and a radioactive assay **(Figs 10A and 1B**) and diminished cell migration in the scratch assay, suggesting its role in making cells unable to repair the damage **(Fig 11A and 11B).**

It has been shown previously that pathogenic effects of *H*. *pylori* LPS were linked to upregulation of gastric mucosal proinflammatory cytokine expression, excessive nitric oxide production, diminished production of regulatory cytokines, and abrogation of cell cycle progression and cell proliferation [45, 81–82]. Li et al. [83] noticed that LPS regulated pro-apoptotic MMP-9 expression through Toll-like (TLR)4/NF-κB signaling, and Cho et al. [84] reported the induction of apoptosis-related genes in human carcinoma cells by *H*. *pylori* VacA toxin [83–84]. This toxin is mainly secreted by *H*. *pylori* to the gastric environment; however, it is also exposed on the surface of bacterial cells and present in the membrane vesicles [76]. Previously, we showed that the proliferation of human peripheral blood lymphocytes was inhibited in the presence of an antigenic complex from *H*. *pylori* G27 VacA+CagA- but not from the G27 VacA-CagA- mutant strain [85]. In the present study, gastric epithelial cells and fibroblasts were stimulated with the glycine acid extract from the *H*. *pylori* CCUG 17874 VacA+CagA+ strain. Therefore, it is possible that VacA could be responsible for certain effects demonstrated in this study. However, further studies are needed to elucidate the involvement of VacA in the regulation of MMP-9 as well as the proliferation and apoptosis of gastric epithelial cells.

In conclusion, the results obtained using an *in vivo* model of guinea pigs experimentally infected with *H*. *pylori* and *in vitro* cellular models of guinea pig primary gastric epithelial cells and fibroblasts indicated that during *H*. *pylori* infection, the soluble virulence factors of these bacteria that are present in a colonization niche can induce cell apoptosis and inhibition of cell proliferation in relation to the inflammatory response and increased oxidative stress. The loss of cells affects the integrity and protective function of the gastric epithelial barrier. *H*. *pylori* components can potentially penetrate the gastric barrier into the basement membrane, where they can affect the pro-regenerative function of fibroblasts supporting the inflammatory response and the development of pathological effects. By crossing these gastric tissue barriers, *H*. *pylori* components can also influence immune cells. However, the results from cellular experiments *in vitro*, in which recombinant proteins are used, should be interpreted with caution. It is not clear whether the effects that they induce are identical to those that are caused by living bacteria.

Our group and other authors have shown previously that some *H*. *pylori* components, including CagA or LPS, have negative immunomodulatory properties, which can facilitate the survival of bacteria and long-lasting infection, thus increasing the risk for ulcer disease and cancer development [20, 23,43, 85–89]. It is worth emphasizing that the effects of individual *H*. *pylori* components may be different. Therefore, considering the island-like nature of *H*. *pylori* infection, it should be assumed that the ratio between the individual components may have a significant impact on their effects in the colonization site. The persistence of the inflammatory reaction together with the simultaneous increase in the proliferation rate of gastric epithelial cells may pose a risk of neoplastic changes, especially in the inflammatory milieu induced by *H*. *pylori* [90]. The translocation of *H*. *pylori* compounds through the digestive tract can potentially induce systemic effects in the host in response to *H*. *pylori* infection.
